# Development of optimal sizing system for tactical gloves applying empirical loss coefficient ratio

**DOI:** 10.1038/s41598-023-38417-x

**Published:** 2023-07-17

**Authors:** Yujin Hong, Hee Eun Choi

**Affiliations:** 1 Department of Fashion and Textiles, Seoul National University, Seoul, 08826 Republic of Korea; 2 Research Institute of Human Ecology, Seoul National University, Seoul, 08826 Republic of Korea

**Keywords:** Health care, Engineering, Materials science, Mathematics and computing

## Abstract

This study aims to improve Korean male soldiers’ mission performance and protect them from safety accidents by establishing an optimal sizing system that considers the fit of tactical gloves and production and supply efficiency. First, the wearing condition of tactical gloves was investigated through in-depth interviews and surveys. The optimal glove fit and loss coefficient ratio was then analyzed through a glove size selection experiment. Finally, the sizing system was optimized and verified by comparing the coverage rate to the current sizing system. The empirically derived loss coefficient ratio was 0.075, and the optimal sizing system for tactical gloves was S-hand length: 168 mm, hand width: 81 mm, M-hand length: 177 mm, hand width: 83 mm, L-hand length: 184 mm, hand width: 86 mm, XL-hand length: 191 mm, and hand width: 89 mm. The coverage rate of the optimal sizing system proposed in this study was 86.4%, showing an improvement of approximately 21.1% compared to the current sizing system (65.3%). In conclusion, the optimal sizing system for tactical gloves proposed in this study can realistically solve current sizing issues, as it improved the coverage rate by 21.1% without incurring additional costs for production or hindering the supply efficiency.

## Introduction

Hand injuries account for one-fifth of all emergencies^1^. This situation is worse in a tactical environment where physical damage such as breakage, cutout, perforation, and shock or thermal risks due to heat and cold frequently occur. Therefore, wearing tactical gloves is essential to prevent primary hand injuries. However, these gloves may sometimes cause safety accidents. Wearing them can prove to be disadvantageous in overall workability, including hand strength, dexterity, task time, and range of motion^2–4^, which can all lead to safety accidents. According to Do^5^, the tactical activities that most frequently caused injuries were mountain rescue (35.29%), helicopter descent (23.53%), and shooting tactics (11.77%), all three of which involve high-level hand activities dealing with equipment such as ropes and firearms. Thus, to ensure the soldier’s overall safety, the trade-off between protective function and task performance of tactical gloves must be overcome.

The most effective solution for overcoming the aforementioned trade-off issue of tactical gloves is to improve the fit by establishing an appropriate sizing system^6^ based on a detailed ergonomic and anthropometric analysis of the hand in motion^7^. However, glove sizing systems proposed in previous studies^8–12^ are unsuitable for wearing tactical gloves in terms of fit, production, and supply efficiency. Sizing systems with a large dimensional interval and a small number of sizes offer advantages in production and distribution efficiency; however, it is highly likely that such gloves will not provide an excellent fit. Conversely, if the dimensional interval is narrow with a large number of sizes, the fit can be improved by providing various sizes to the wearer; however, it is not a realistic solution given its excessive production cost^13^.

This study applied the concept of loss function to develop a sizing system that can provide the best fit for the gloves of male Korean soldiers. The loss function is a mathematical model for production size optimization proposed by Taguchi^14^, which defines the loss as the gap between the consumer’s required dimension and the provided product dimension. This model has been adjusted based on the unique characteristic of apparel products by Park and Kim^15^ and used in sizing systems for various items. However, it has not been applied to the development of a sizing system for gloves. Additionally, given that the loss coefficient ratio put into the loss function in previous studies^13,16–19^ was established based on the researcher’s intuition or approximation according to the feature of each item, an objective research method is required to derive the loss coefficient ratio for gloves.

Thus, this study analyzed the optimal fit of the tactical gloves under various tactical posture conditions and applied it to the concept of loss function optimization, thereby developing a new sizing system for both the fit of gloves and the efficiency of production and supply. For the efficiency of production and supply, the current number of sizes is maintained. We presented non-uniform optimal dimensional intervals to improve the fit and cover rate of the tactical gloves. Additionally, we adopted a new empirical approach to derive a loss coefficient ratio in the size system optimization process and applied it as a glove size boundary. Thus, we aimed to develop and propose an empirical approach to the sizing system of tactical gloves suitable for tactical activities, thereby improving their fit to help meet mission performance and physical protection.

## Methods

This study consists of four stages: (1) investigation of the wearing condition of tactical gloves through in-depth interviews and surveys; (2) analysis of the glove fit and loss coefficient ratio through an experiment; (3) optimization of the sizing system; and (4) verification of the optimal sizing system by comparing the coverage rate to the current sizing system (Table 1). The interviews, surveys, experiment, and analysis of 8th Size Korea data in this study were carried out with the approval of the Seoul National University Institutional Review Board (SNUIRB; IRB No. 2212/002-002, IRB No. E2211/002-006). All methods were conducted in accordance with relevant guidelines and regulations. Informed consent was obtained from all participants regarding including their information/images in an online open-access publication and paper. We confirmed that informed consent for participation was obtained from all participants prior to all interviews, surveys, and experiments. This experiment faithfully followed the strict regulations and guidelines of the Institutional Review Board.

**Table 1:** Research procedure.

Stages	Contents	
Stage 1	Investigation of the wearing condition	In-depth interview (n = 4)
		Survey (n = 104)
Stage 2	Analysis of the optimal fit and loss coefficient ratio	Experimental glove design
		Experiment (n = 30)
		Data analysis
Stage 3	Optimization of the sizing system	
Stage 4	Verification of the optimal sizing system	

### Investigation of the wearing condition

#### In-depth interview

In-depth interviews were conducted to identify problems with the current tactical gloves and to design survey questions. Participants included four adult men (two in the Army, one in the Air Force, and one in the Navy) who had served in the Korean military within the last 5 years. The first draft of the questionnaire was designed by reflecting on the participants’ responses, and the final questionnaire was completed after review and revision by the same interview participants.

#### Survey

We administered a survey to 104 adult men in their 20 s and 30 s who had previously experienced wearing tactical gloves during their military service to understand the wearing condition of tactical gloves. The survey comprised eight questions, including two on demographic information, two on general information about military service, and four on the type of tactical gloves worn during military service and how these gloves are worn. The response results were analyzed through descriptive statistics, frequency analysis, and multiple response analysis by IBM SPSS 26.0.

### Analysis of the optimal fit and loss coefficient ratio

#### Experimental glove design

**Figure 1:**
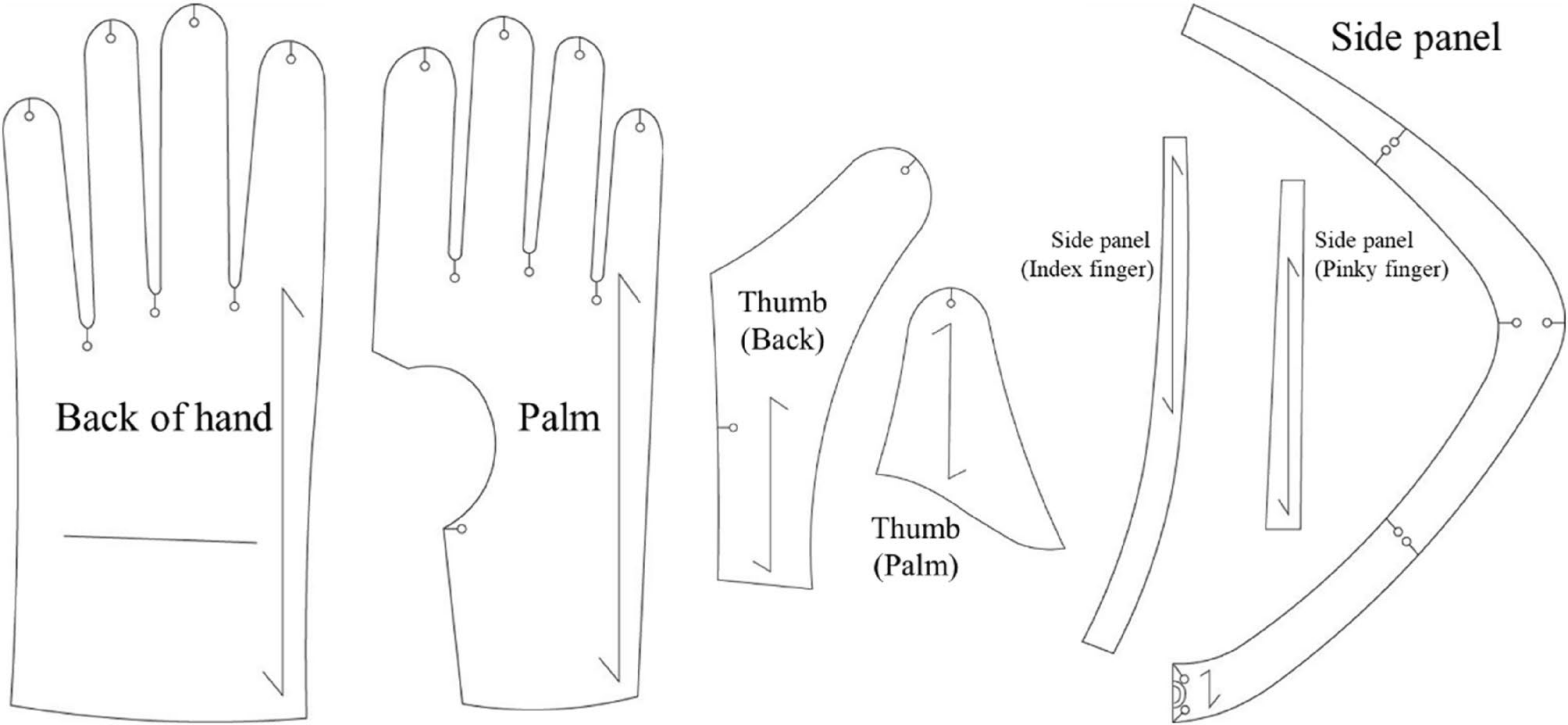
Pattern of experimental gloves.

Based on the survey results, the experimental gloves were developed using the tactical gloves that the majority of respondents wore during military service as a model. To ensure applicability to the Korean military, the pattern, materials, and grading rule of the experimental gloves were designed to match those of current tactical gloves. The original pattern of the current tactical glove includes seven pieces of side panels and attached panels to prevent injury. However, to minimize the effect of unnecessary seam lines on the experimental results, the experimental glove pattern was adjusted by simplifying the side panel into three pieces and removing the attached panels (Fig. 1). The current tactical gloves are made of two different materials: knitted fabric and leather. Therefore, two types of fabric with material properties similar to those of the current tactical gloves were selected as the material for the experimental gloves (Table 2). The size interval between the glove length and width was equally graded to 2 mm, and the grading ratio between items was similar to the ratio of the current tactical gloves (Table 3).

**Table 2:** Physical properties of experimental gloves materials.

Item	Type	Composition	Weight (g/m^2^)	Thickness (mm)	Tensile strength (N/mm^2^)	Elongation (%)
Fabric 1	Jersey	Cotton 50%, Polyester 50%	309.33	1.23	Wale: 0.09	Wale: 64.51
					Course: 0.12	Course: 198.54
Fabric 2	Artificial leather	Polyurethane 65%, Rayon 35%	373.33	0.72	Warp: 0.09	Warp: 21.145
					Filling: 0.05	Filling: 31.71

**Table 3:** Size of the experimental gloves (Unit: mm).

Size	GL	GW	Size	GL	GW	Size	GL	GW
1	226	93	7	238	105	13	250	117
2	228	95	8	240	107	14	252	119
3	230	97	9	242	109	15	254	121
4	232	99	10	244	111	16	256	123
5	234	101	11	246	113	17	258	125
6	236	103	12	248	115	18	260	127

GL, glove length; GW, glove width.

#### Experiment

The experimental participants included 30 healthy adult men in their 20 s and 30 s who participated in tactical training during their military service (Table 4). The sample group was chosen because this experiment required various hand movements, such as shooting and rope postures, that are performed during tactical activities.

**Table 4:** Experiment participants’ characteristics (n = 30).

	Min	Max	Mean (SD)
Age	22	38	28 (4)
Hand length (mm)	178	205	191 (7)
Hand width (mm)	85	97	90 (3)

Based on the survey results, hand movements (shooting posture, rope posture) and hand function (dexterity, grip strength) required in the noted tactical situation were selected as experiment conditions for analyzing the optimal fit and computing the loss coefficient ratio.

In step 1, hand length, hand width, and the lengths of the five fingers of each participant were measured. In step 2, the participants selected the subjective suitable glove (${SG}$) and the allowable glove range (${AG}_{Range}$). They wore 18 sizes of experimental gloves in a natural, static position, and chose the $SG$ that best fit their hands. Then, they tried smaller and larger gloves than their chosen $SG$, selecting the minimum and maximum allowable glove ($AG_{min}$ and $AG_{Max}$). $SG$, $AG_{min}$, and $AG_{Max}$ were also selected in shooting and rope postures, respectively, in the same way as in a static posture. In step 3, dexterity and grip strength were measured to derive the objective ${AG}_{Range}$. The hand dexterity was measured in seven different glove size wearing conditions, from gloves three sizes smaller than ${SG}_{static}$ to those three sizes larger than ${SG}_{static}$, via the Purdue pegboard test. Using a dynamometer, we also measured the grip strength in 11 different glove size wearing conditions, from gloves five sizes smaller than ${SG}_{static}$ to those five sizes larger than ${SG}_{static}$.

#### Data analysis

To analyze the optimal fit of tactical gloves, changes in subjective $SG$, $AG_{min}$, and $AG_{Max}$ between the static and dynamic postures were statistically analyzed through the paired sample t-test of IBM SPSS 26.0. The items whose significant changes were confirmed were analyzed in detail through the results of the in-depth interview conducted within the experiment. We also derived objective ${AG}_{min}$ and ${AG}_{Max}$ by analyzing the dexterity and grip strength test records. To exclude each participant’s natural physical ability and compare the test records according to only the size of the glove, we analyzed the extent of change in the test records based on the test record of ${SG}_{static}$.

We calculated the final $AG$ range through the intersection of the subjective $AG$ range in static, shooting, and rope postures and the objective $AG$ range according to the dexterity and grip strength tests. The loss coefficient ratio ($C_{2} /C_{1}$) used in the optimization process was derived through the equation of $C_{2} /C_{1} = \left( {AG_{min} /AG_{Max} } \right)^{2}$ according to Park and Kim^15^.

### Optimization of the sizing system

The development of the tactical glove size system in this study was carried out through the optimization of the data-driven method based on the loss function. The loss function $L(x)$ is a model for production size optimization proposed by Taguchi^14^, which defines the loss as the difference between the consumer’s required dimension ($x$) and the provided product dimension ($u$) (1). By applying the Taylor expansion (2), the $L(x)$ can be approximated as shown in (3).

1 $$ L\left( x \right) = f\left( {x - u} \right) $$

2 $$ L\left( x \right) = L\left( u \right) + \frac{{L^{\prime}\left( x \right)}}{1!}\left( {x - u} \right) + \frac{{L^{\prime\prime}\left( x \right)}}{2!}(x - u)^{2} + \frac{{L^{\prime\prime\prime}\left( x \right)}}{3!}(x - u)^{3} + \cdot \cdot \cdot $$

3 $$ L\left( x \right) \approx C(x - u)^{2} $$

Later, Park and Kim^15^ proposed that, in the case of apparel products, the probability of the wearer choosing a smaller or larger size will vary depending on the wearer’s preference, wearing purpose, and wearing environment. Accordingly, the loss function was adjusted by splitting the loss coefficient based on the two conditions of selecting a smaller and a larger size (4).

4 $$ L\left( x \right) = \left\{ {\begin{array}{*{20}c} {C_{1} (u - x)^{2} , \; if \;x \le u} \\ {C_{2} (x - u)^{2} , \;if\; x > u } \\ \end{array} } \right. $$

The goal of this study is to optimize the size by minimizing the sum of all wearers’ $L(x)$. The sizing system optimization of tactical gloves was performed by inputting the hand length and width data of 1,341 adult men in their 20 s and 30 s, collected through the 8th Size Korea. The optimization was performed under the condition of one size to seven sizes, respectively, and the final number of sizes was determined by considering the total loss and the efficiency of production and supply.

### Verification of the sizing system

To verify the optimal sizing system for tactical gloves proposed in this study, the coverage ratio was compared to the current sizing system using IBM SPSS 26.0 cross-analysis. The current sizing system consists of four sizes: S, M, L, and XL, and the length and width of the gloves are deployed at intervals of 5 mm (Table 5). However, only the product dimensions are presented accordingly; the body dimensions corresponding to each size were not specified. Therefore, in this study, the hand length for each size was estimated because the gap between the wrist line and the hem line was 60 mm in the pattern of the current tactical gloves. Furthermore, data obtained from the 8th Size Korea were analyzed to obtain the hand width corresponding to the hand length of the M size; based on these data, the hand width dimensions of the current sizing system were derived by applying the 5 mm intervals.

**Table 5:** Current sizing system of tactical gloves (Unit: mm).

	S	M	L	XL
Glove length	235 $\pm$ 3	240 $\pm$ 3	245 $\pm$ 3	250 $\pm$ 3
Glove width	100–104	105–109	110–114	115–119
Hand length	175	180	185	190
Hand width	80	85	90	95

## Results and discussion

### Investigation on the wearing condition

By comparing the response percentage of 12 different tactical activities where the tactical gloves were recommended to be worn and the activities where the gloves were actually worn, the gap between recommended wear and actual wear was revealed to be higher than 15% in the shooting and rope descending activities. The survey respondents additionally demonstrated that wearing tactical gloves is highly recommended to prevent hand injuries and chilblains; however, soldiers do not prefer wearing them because they interfere with hand movement and sensation, particularly when controlling firearms or grabbing a rope. Therefore, two hand movements (shooting and rope postures) and two hand functions (dexterity and grip strength) required for military activities were selected as experimental items to analyze the optimal fit and loss coefficient ratio.

### Analysis of the optimal fit and loss coefficient ratio

#### Subjective $SG$ and ${AG}_{Range}$

The mean (SD) value of $SG$, $AG_{min}$, and $AG_{Max}$ in static, shooting, and rope postures are shown in Table 6.

**Table 6:** Mean (SD) value of $SG$, $AG_{min}$, and $AG_{Max}$ in each posture (Unit: size).

		${AG}_{min}$	$SG$	${AG}_{Max}$
Static posture		2.77 (1.583)	6.46 (2.158)	10.42 (2.301)
Dynamic posture	Shooting posture	2.81 (1.980)	5.35 (2.770)	9.04 (3.256)
	Rope posture	2.00 (1.549)	4.12 (2.304)	7.81 (2.546)

Paired sample t-tests were performed to statistically analyze the optimal fit of tactical gloves by analyzing the changes in the $SG$, $AG_{min}$, and $AG_{Max}$ between static and dynamic postures (Table 7).

**Table 7:** Changes in the $SG$, $AG_{min}$, $AG_{Max}$ between static and dynamic postures.

	Static posture (Unit: size)	Dynamic posture (Unit: size)		t	p
${AG}_{min}$	2.77	Shooting posture	2.81	− 0.135	0.894
		Rope posture	2.00	3.077	0.005**
$SG$	6.46	Shooting posture	5.35	3.248	0.003**
		Rope posture	4.12	7.604	0.000***
${AG}_{Max}$	10.42	Shooting posture	9.04	2.807	0.010**
		Rope posture	7.81	7.023	0.000***

***p < 0.001, **p < 0.01.

**Figure 2:**
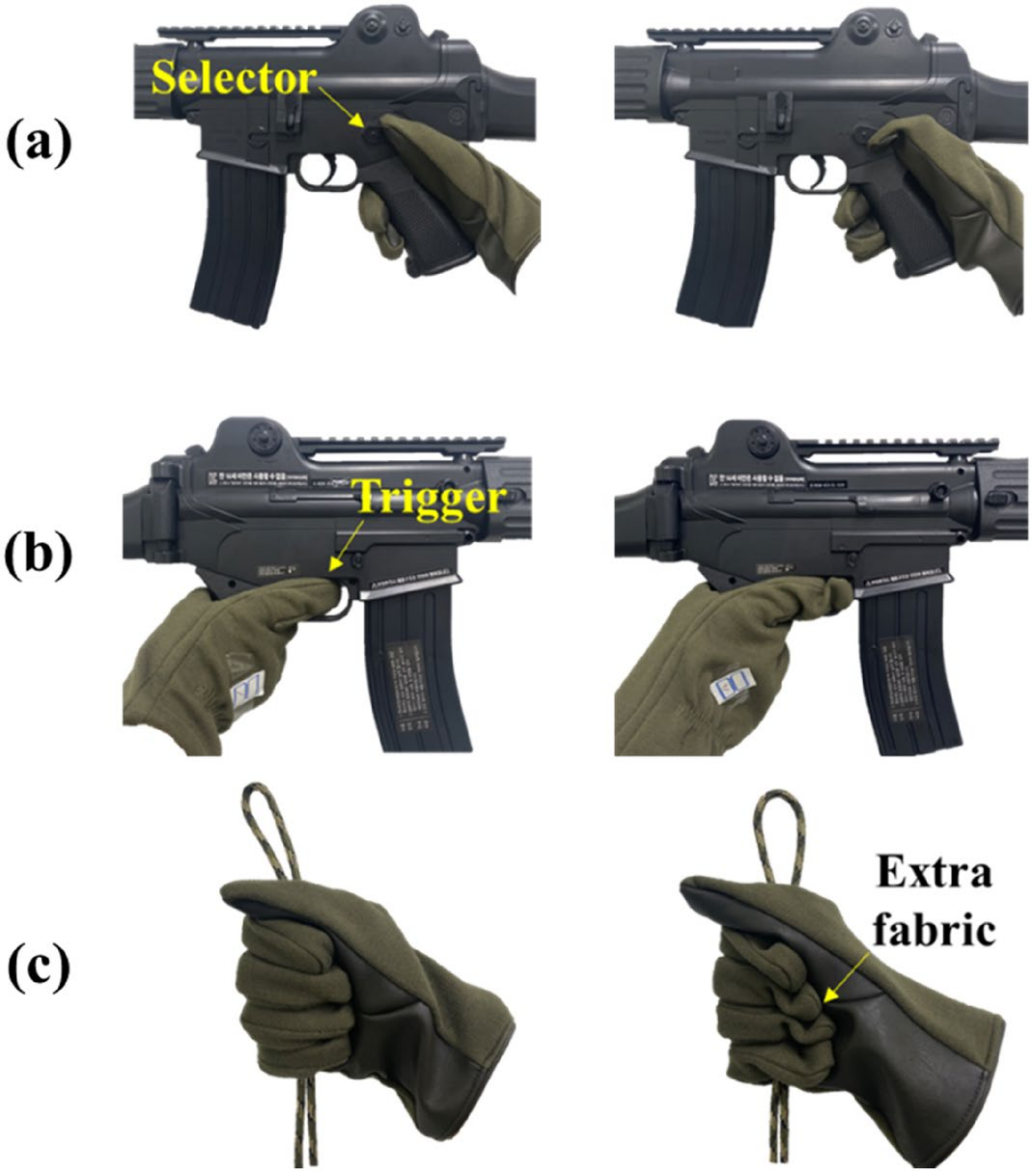
Difficulties in tactical hand postures when wearing large gloves. (**a**) Control of selector (left: SG, right: large glove), (**b**) control of trigger (left: SG, right: large glove), (**c**) grasping rope (left: SG, right: large glove).

According to the results, the ${AG}_{min}$ in static and shooting postures showed no statistically significant change, but the $SG$ and ${AG}_{Max}$ showed a significant decrease at the 0.01 level. During the in-depth interviews, the participants demonstrated that they prefer tight gloves in shooting postures because large and loose-fitted gloves make it difficult for the thumb to control the firearm selector and for the index finger to pull the trigger (as shown in Fig. 2a and b), which increases anxiety about safety accidents such as accidental discharge.

Additionally, $SG$, $AG_{min}$, $AG_{Max}$ in static and rope postures showed a statistically significant decrease. This occurred due to the need for additional strength in the hand and high physical strength consumption as the extra fabric of the loose-fitted gloves interfered with the grasping posture and caused unexpected sliding (Fig. 2c).

#### Objective ${AG}_{Range}$

**Figure 3:**
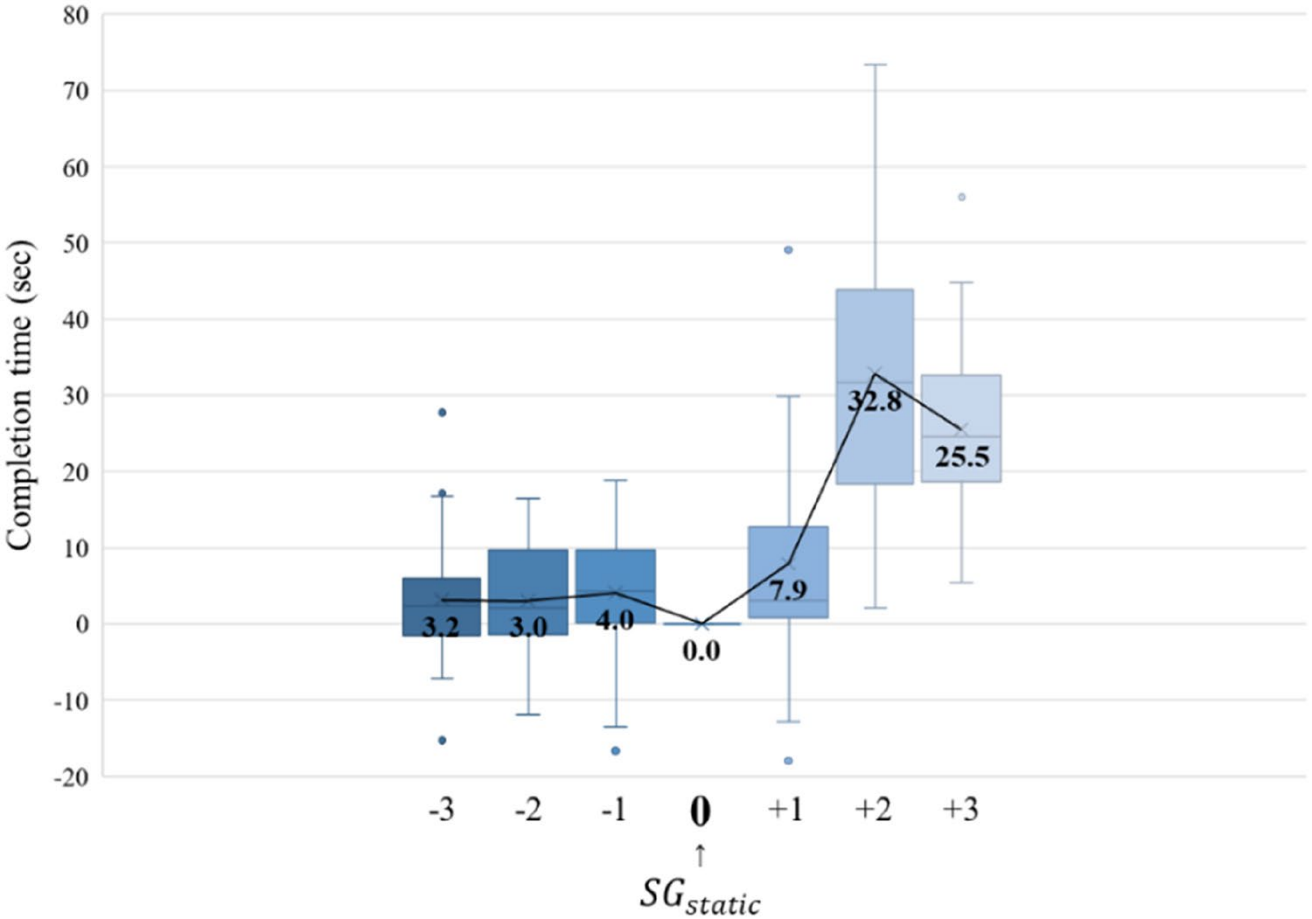
Results of dexterity tests.

The dexterity tests (Fig. 3) showed a difference in the record within 10 s, from a glove that was three sizes smaller (− 3) to a glove that was one size larger (+ 1) based on the $SG$. Thus, it did not seem to have a significant impact on the dexterity. However, a glove two (+ 2) and three (+ 3) sizes larger showed a record difference of more than 25 s, which significantly affected the degradation of dexterity. Therefore, the ${AG}_{min}$ was recorded to be − 3 or less and the ${AG}_{Max}$ was + 1 according to the dexterity tests.

**Figure 4:**
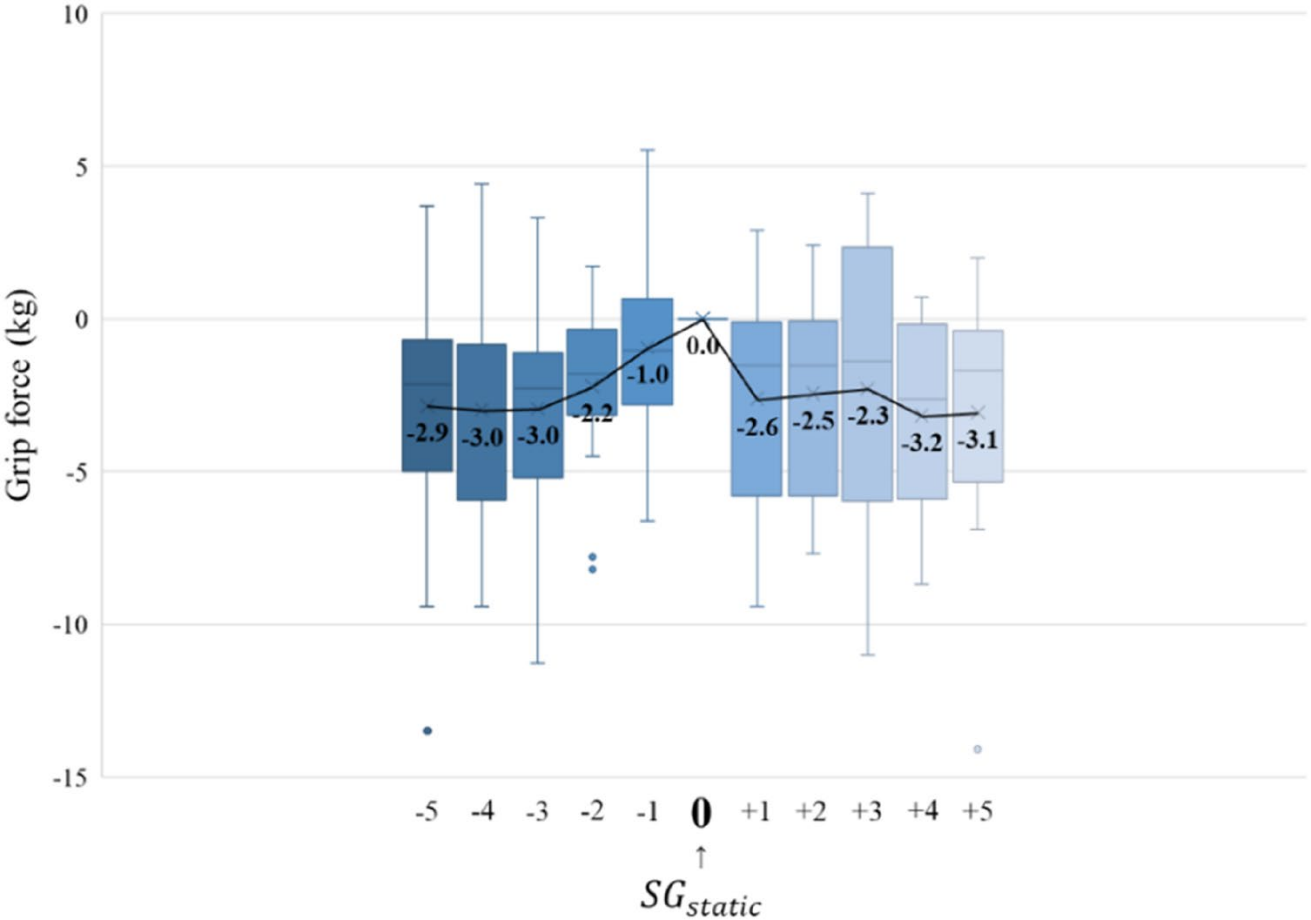
Results of grip strength tests.

The grip strength tests (Fig. 4) revealed that all the records showed a slight decrease within 3.2 kg. Therefore, no significant decrease in grip strength occurred depending on the size of the gloves, and it was predicted that the ${AG}_{min}$ and ${AG}_{Max}$ would occur below -5 and above + 5, respectively.

#### Final ${AG}_{Range}$ and loss coefficient ratio

**Figure 5:**
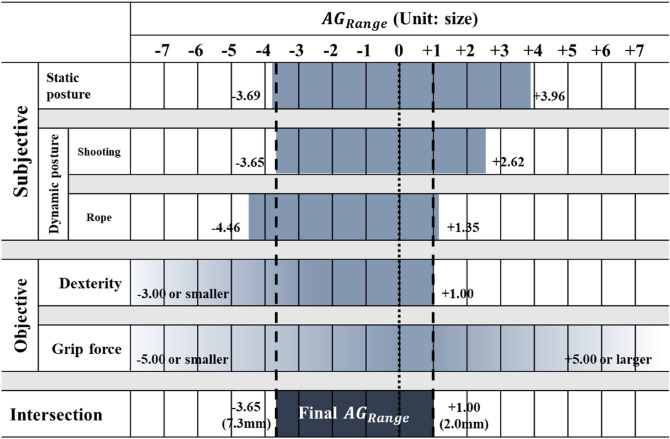
The final $${AG}_{Range}$$.

The final ${AG}_{Range}$ was calculated in the range 3.65 size smaller than $SG$ to 1 size larger than $SG$ by the intersections of each ${AG}_{Range}$ of five experimental conditions (Fig. 5). After converting this into mm, the final ${AG}_{Range}$ is in the range of the 7.3 mm smaller glove to the 2.0 mm larger glove. The loss coefficient ratio (${C}_{2}/{C}_{1}$) used in the optimization process can be obtained through the equation of ${C}_{2}/{C}_{1}=({AG}_{min}/{AG}_{Max}{)}^{2}$ according to Park and Kim^15^. Therefore, the result of the loss coefficient ratio derived in this study is 0.075 (5).

5 $$ C_{2} /C_{1} = (\beta /\alpha )^{2} = \left( {\frac{2mm}{{7.3mm}}} \right)^{2} \approx 0.075 $$

Results obtained in this study show the opposite aspect of the loss coefficient ratio obtained in previous studies that had applied the concept of the loss function to apparel sizing systems. In previous studies, the loss coefficient ratio was set to a constant greater than 1, assuming that the loss is larger when selecting a small size compared to selecting a large size, or set to 1, assuming that the loss is equal in both choices^13,16–19^. Contrarily, the loss coefficient ratio derived in this study is a constant of less than 1, which means that the loss is significantly smaller when selecting a small size compared to selecting a large size.

Loss caused by wearing improper-sized tactical gloves is directly related to life. In particular, the results of the in-depth interview show that wearers of tactical gloves experienced anxiety and difficulty in controlling tactical equipment when wearing large gloves. We can interpret these results to mean that wearers think it is more important to wear smaller gloves to secure the convenience and safety of work rather than experience the comfort of wearing large gloves.

### Optimization of the sizing system

#### Optimal dimension computation

**Figure 6:**
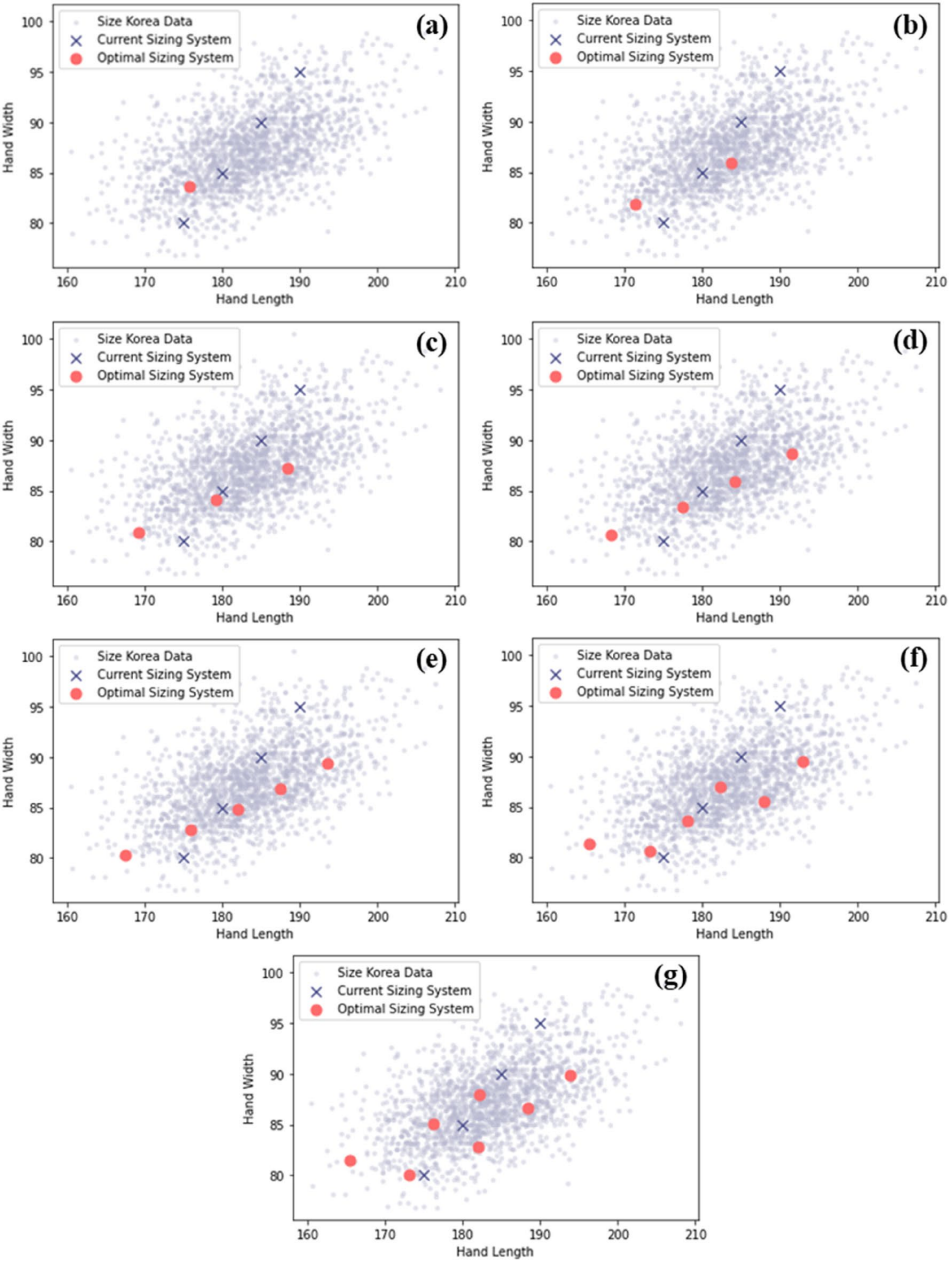
Optimization results. (**a**) One size, (**b**) two sizes, (**c**) three sizes, (**d**) four sizes, (**e**) five sizes, (**f**) six sizes, (**g**) seven sizes.

Figure 6 shows the optimization results according to the number of sizes. In the optimization results, the optimal dimension was located on the relatively small side compared to the distribution of the total hand length and width. This can be seen as a result that reflects higher tolerance characteristics for gloves of smaller sizes than that for gloves of larger sizes.

**Figure 7:**
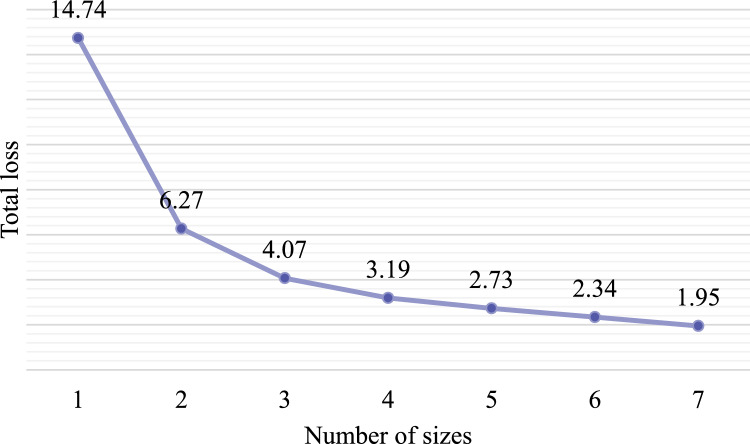
Changes in total loss with number of sizes.

Total loss decreased as the number of sizes increased (Fig. 7). When the number of sizes changed from one to two, the total loss decreased significantly; however, after four or more sizes, the total loss decreased within 0.5 and maintained a similar level. Therefore, four sizes applied in the current sizing system were preserved in this study to take advantage of the small loss and avoid incurring additional costs for production or hindering the efficiency of supply. Table 8 shows the results of the optimal dimensions by setting the number of sizes to four.

**Table 8:** Optimal dimension under four sizes condition (unit: mm).

	${u}_{1}$	${u}_{2}$	${u}_{3}$	${u}_{4}$
Hand length	168.31	177.43	184.12	191.48
Hand width	80.57	83.40	85.93	88.71

$$u$$, optimal dimension.

#### Boundary construction and overlapping section definition

**Figure 8:**
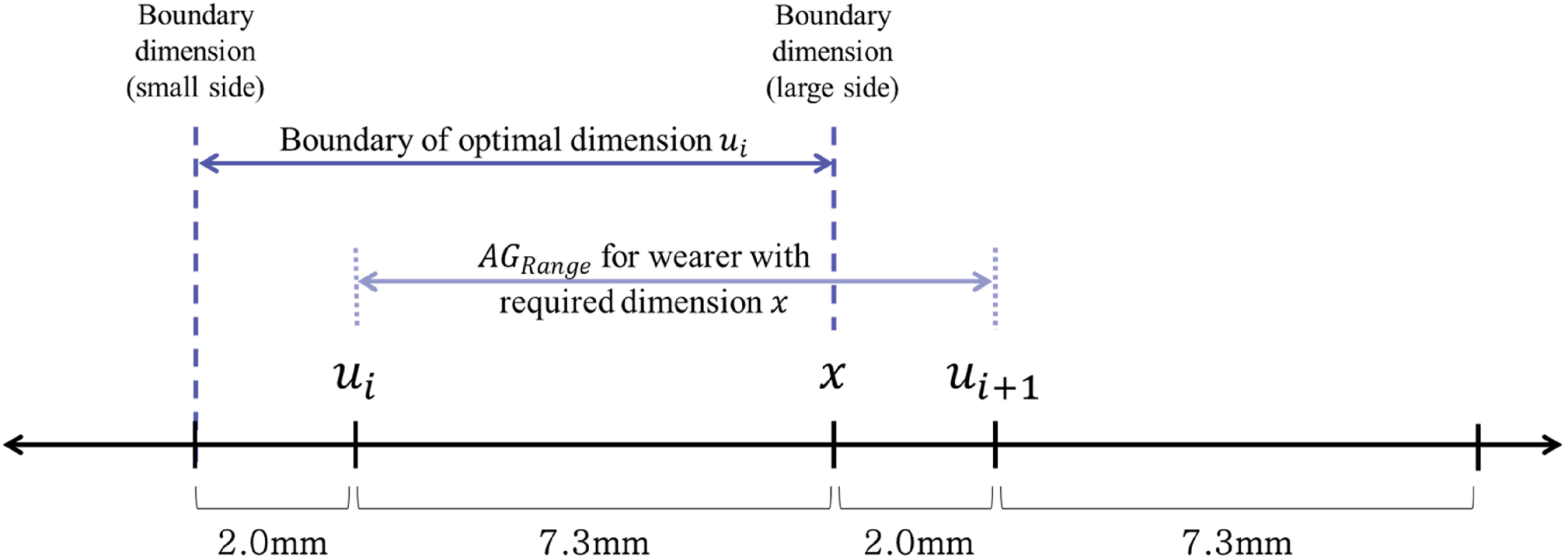
Relationship between the ${AG}_{Range}$ and the boundary of the optimal dimension.

The final ${AG}_{Range}$ considering the tactical activities is from − 7.3 to + 2.0 mm based on $SG$ (Fig. 5). Thus, as shown in Fig. 8, a wearer with the required dimension (human body dimension) of $x$ can wear sizes ranging from an optimal dimension ${u}_{i}$, that is, 7.3 mm smaller than his or her required dimension, to an optimal dimension ${u}_{i+1}$, that is, 2.0 mm larger than the required dimension. If this is interpreted from the perspective of optimal dimension rather than the wearer’s point of view, the optimal product dimension ${u}_{i}$ can be worn by a wearer with a 2.0 mm smaller dimension to a wearer with a 7.3 mm larger dimension.

**Figure 9:**
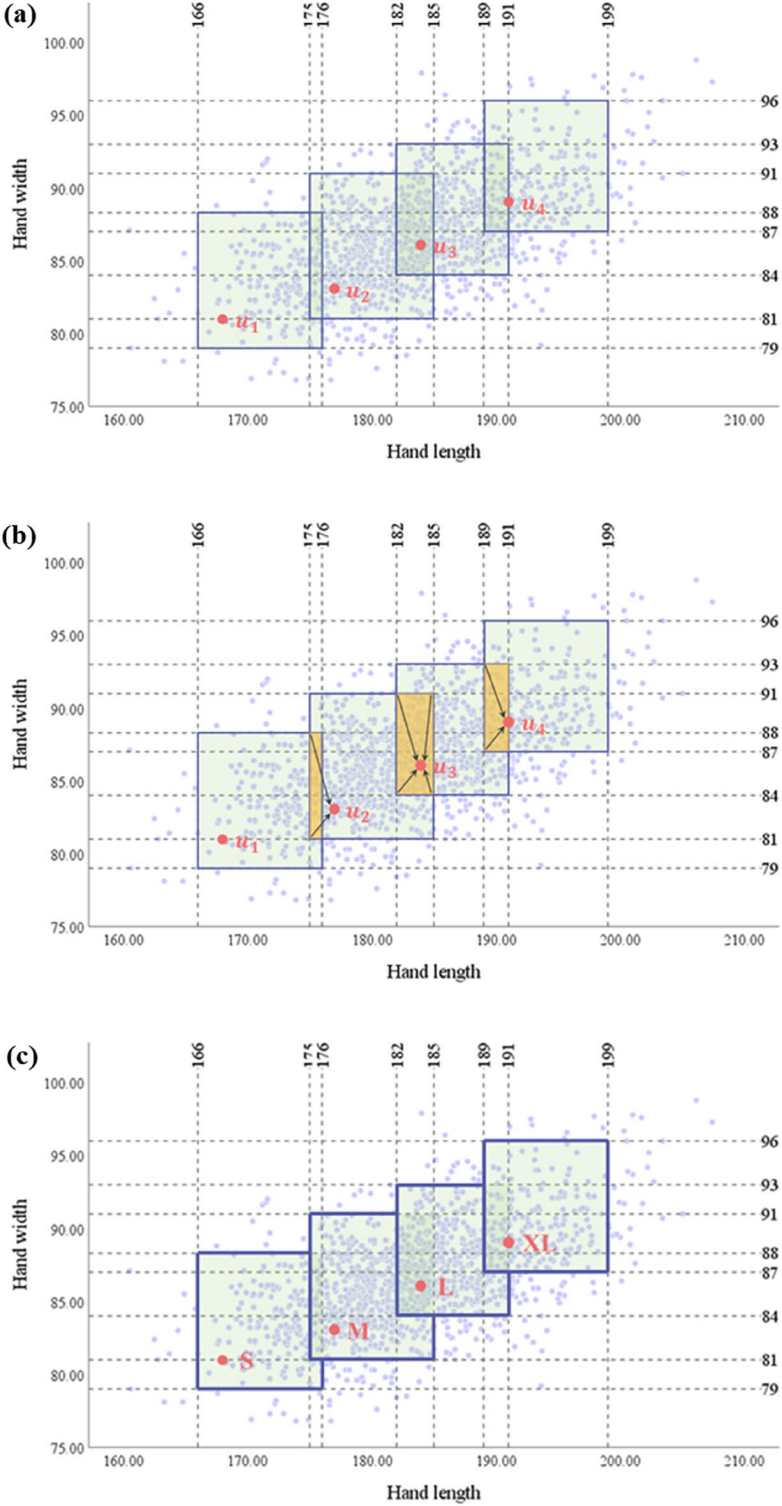
Development of optimal sizing system for tactical gloves (Unit: mm). (**a**) Optimal dimensions and boundaries applied to 8th Size Korea data, (**b**) size definition of overlapping section, (**c**) optimal sizing system of tactical gloves.

Accordingly, the boundary from − 2.0 mm to + 7.3 mm was applied to the optimal dimensions of the hand length and hand width in Table 9. The optimal dimensions and boundaries were rounded from the first decimal place, and only natural numbers were presented for convenience. They were also applied to the distribution of hand length and hand width data of adult men in their 20 s and 30 s collected through the 8th Size Korea (Fig. 9a), and the overlapping section was defined as the size of the nearest optimal dimension (Fig. 9b). Thus, the final optimal sizing system for tactical gloves were decided as shown in Fig. 9c.

**Table 9:** Optimal dimensions and boundaries of hand length width (Unit: mm).

		${u}_{1}$		${u}_{2}$		${u}_{3}$		${u}_{4}$	
Hand length	Optimal dimension	168		177		184		191	
	Boundary	166	176	175	185	182	191	189	199
Hand width	Optimal dimension	81		83		86		89	
	Boundary	79	88	81	91	84	93	87	96

### Verification of the sizing system

**Figure 10:**
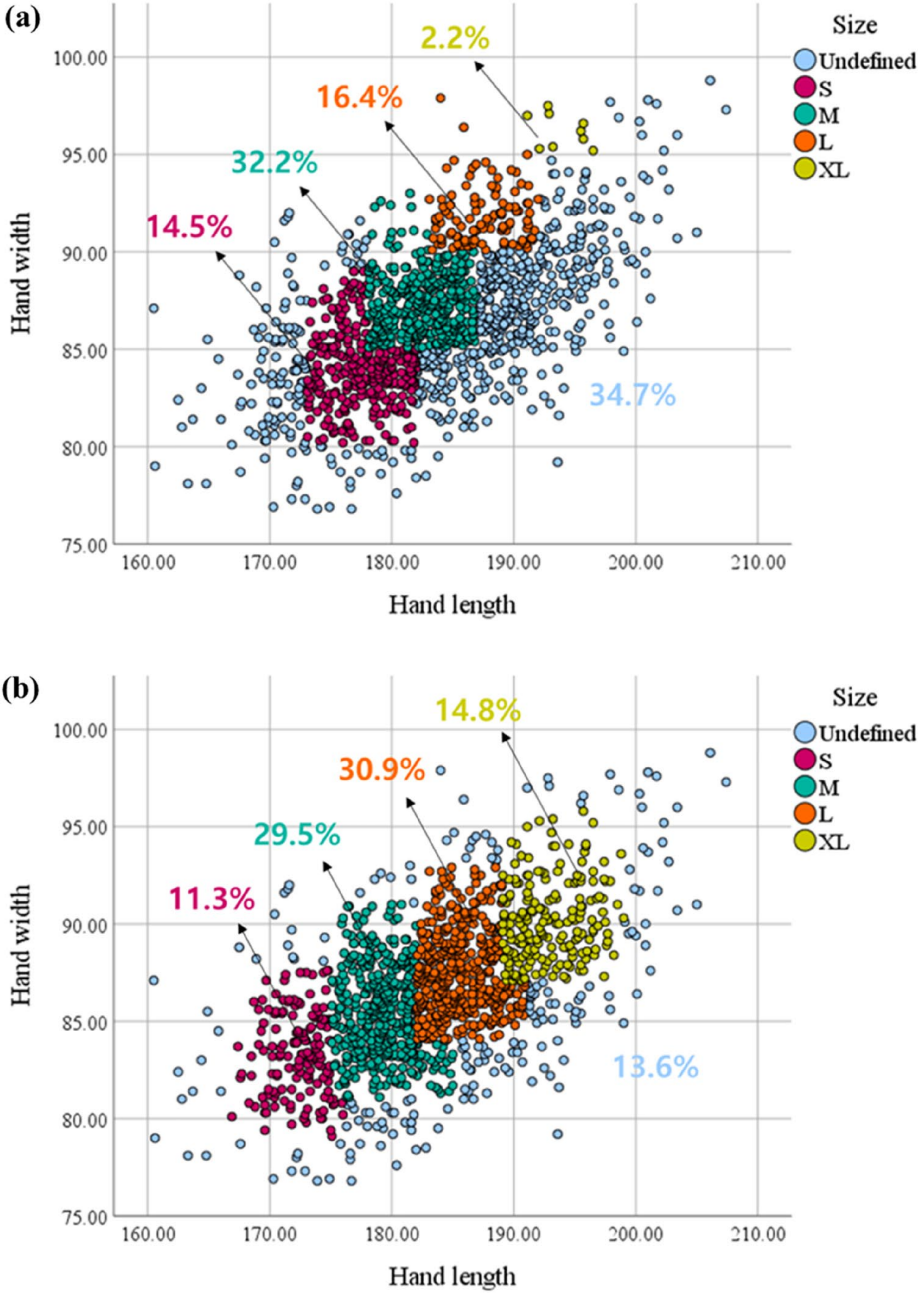
Comparison of coverage rate (Unit: mm). (**a**) Coverage rate of the current sizing system, (**b**) coverage rate of the optimal sizing system.

To compare the coverage rate, the boundary construction and overlapping section definition were applied to the current sizing system using the same method as the optimal sizing system. The coverage rate of the current sizing system and optimal sizing system were calculated to be 65.3% and 86.4%, respectively, based on the 8th Size Korea data (Fig. 10).

## Conclusion

The purpose of this study was to improve Korean male soldiers’ mission performance and avoid safety accidents by establishing an optimal sizing system that considers both the fit of tactical gloves and the efficiency of production and supply. Accordingly, this study conducted an in-depth interview and survey on the wearing of tactical gloves, analyzed the optimal fit of tactical gloves through experiments, derived the loss coefficient ratio, and developed an optimal sizing system for tactical gloves.

The survey results showed that the percentage gap between recommended and actual wearing was higher than 15% in shooting and rope descending among 12 tactical activities. Thus, hand movements (shooting posture, rope posture) and hand functions (dexterity, grip strength) required in shooting and rope descent were selected as experimental items to analyze the optimal fit of tactical gloves and derive the loss coefficient ratio.

The optimal fit of tactical gloves was analyzed through changes in $SG$, $AG_{min}$, and $AG_{Max}$ between static and dynamic postures. The results showed a statistically significant decrease in $SG$ and $AG_{Max}$ between static and shooting positions, due to difficulty in controlling the firearm, and anxiety about safety accidents due to malfunctioning firearms. Additionally, $SG$, $AG_{min}$, and $AG_{Max}$ showed statistically significant reductions due to the need for additional strength and high physical strength in the hand due to the extra fabric of the loose-fitted gloves interfering with the grasping posture.

The loss coefficient ratio of tactical gloves was derived through the intersection of ${AG}_{Range}$ to satisfy the static, shooting, and rope postures, dexterity, and grip strength conditions. The final ${AG}_{Range}$ was derived from − 7.3 to + 2.0 mm based on ${SG}_{static}$, and the loss coefficient ratio was calculated to be 0.075. This result contrasts with previous studies that have applied the loss coefficient ratio to a constant greater than 1, assuming that the loss is greater when selecting a small size compared to selecting a large size, or set to 1, assuming that the loss is equal in both choices. This is because tactical glove wearers favor the advantages of wearing small gloves, such as ease and safety during tactical activities, more than the comfort of wearing large gloves.

The sizing system of tactical gloves was optimized by applying the 8th Size Korea data of 1,341 adult men and the loss coefficient ratio calculated through the experiment. The optimized dimensions of four sizes were S-hand length: 168 mm, hand width: 81 mm; M-hand length: 177 mm, hand width: 83 mm; L-hand length: 184 mm, hand width: 86 mm; XL-hand length: 191 mm, hand width: 89 mm.

The optimal sizing system for tactical gloves was verified by comparing it with the current sizing system in terms of coverage rate. As a result, the coverage rate of the optimal sizing system proposed in this study was 86.4%, showing an improvement of about 21.1%, while the coverage rate of the current sizing system was 65.3%.

The academic implication of this study is that it reduced the loss in terms of the wearer’s fit by setting uneven dimensional intervals through size system optimization, whereas the current sizing system uniformly divided the size into 5 mm units. Additionally, while previous studies relied on the researcher’s intuition or approximation for determining the loss coefficient ratio used in the loss function optimization process, this study is significant in that it devised a method to derive the empirical value of the loss coefficient ratio through experiments. Additionally, the loss coefficient ratio of tactical gloves derived in this study is less than 1, which indicates that functional gloves, such as tactical gloves, may be preferred in smaller sizes that allow work efficiency and safety.

The practical implication of the optimal sizing system for tactical gloves proposed in this study is that it was developed based on the hand movements and functions required in tactical activities, such as shooting posture, rope posture, dexterity, and grip strength. Unlike the current sizing system that is based only the product dimensions of tactical gloves, the optimal sizing system proposed in this study can be an effective reference for soldiers to choose gloves by employing actual body dimensions. Additionally, the optimal sizing system for tactical gloves proposed in this study can be a realistic solution to current sizing problems. This is because it improves the coverage rate by 21.1% without incurring additional costs for production or hindering the efficiency of supply by maintaining the number of sizes of the current system.

The optimal sizing system for tactical gloves proposed in this study was proven to be more effective than the current sizing system in terms of coverage rate. However, additional empirical verification through actual wearability evaluation should be conducted. Thus, as a follow-up study, tactical gloves according to the optimal sizing system should be produced in real life and compared with current tactical gloves. Additionally, the wearability of tactical gloves should be reviewed, not only in terms of the sizing system, but also in terms of pattern and design. These aspects can be improved in follow-up studies. Furthermore, we also suggest experiments involving a wider population beyond occupational groups can be conducted to enhance the generalizability of the findings.

## Data Availability

The in-depth interview, survey, and experiment datasets generated and analyzed during the current study are available from the corresponding author on reasonable request. The hand length and hand width data that support the findings of this study are available from Size Korea. However, restrictions apply to the availability of these data. The data were used under license for the current study and are not publicly available. The data can be made available from the authors upon reasonable request and with permission of Size Korea.
